# On the small angle twist sub-grain boundaries in Ti_3_AlC_2_

**DOI:** 10.1038/srep23943

**Published:** 2016-04-01

**Authors:** Hui Zhang, Chao Zhang, Tao Hu, Xun Zhan, Xiaohui Wang, Yanchun Zhou

**Affiliations:** 1 Shenyang National Laboratory for Materials Science, Institute of Metal Research, Chinese Academy of Sciences, 72 Wenhua Road, Shenyang 110016, China; 2 University of Chinese Academy of Sciences, Beijing 100049, China; 3 Department of Materials Science and Engineering, Case Western Reserve University, 10900 Euclid Ave, Cleveland, OH, 44106, USA; 4 Science and Technology on Advanced Functional Composite Laboratory, Aerospace Research Institute of Materials & Processing Technology, No. 1 South Dahongmen Road, Beijing 100076, China

## Abstract

Tilt-dominated grain boundaries have been investigated in depth in the deformation of MAX phases. In stark contrast, another important type of grain boundaries, twist grain boundaries, have long been overlooked. Here, we report on the observation of small angle twist sub-grain boundaries in a typical MAX phase Ti_3_AlC_2_ compressed at 1200 °C, which comprise hexagonal screw dislocation networks formed by basal dislocation reactions. By first-principles investigations on atomic-scale deformation and general stacking fault energy landscapes, it is unequivocally demonstrated that the twist sub-grain boundaries are most likely located between Al and Ti4*f* (Ti located at the 4*f* Wyckoff sites of *P*6_3_/*mmc*) layers, with breaking of the weakly bonded Al–Ti4*f*. The twist angle increases with the increase of deformation and is estimated to be around 0.5° for a deformation of 26%. This work may shed light on sub-grain boundaries of MAX phases, and provide fundamental information for future atomic-scale simulations.

Ti_3_AlC_2_ is an essentially important member in the family of machinable layered ternary carbides and nitrides^1^,^2^, whose chemical formula can be generalized as M_*n*+1_AX_*n*_ (referred to as MAX phases; M is an early transition metal element; A is an A group element; X is C or N; *n* is an integer)^1^,^2^,^3^,^4^,^5^,^6^,^7^. Combining the merits of ceramics and metals, MAX phases are of vital technological importance^2^,^4^,^5^,^6^. Like ceramics, they exhibit high elastic stiffness and strength, good oxidation and corrosion resistance; like metals, they have high electrical and thermal conductivity, excellent machinability and thermal shock resistance^1^,^2^,^3^,^4^,^5^,^6^,^7^.

In the deformation of MAX phases, dislocations play a crucial role. The dislocations in uniaxially deformed MAX phases are predominantly confined in the basal plane^8^,^9^,^10^,^11^,^12^,^13^. Out-of-basal-plane dislocations have only been observed in nanoindented Ti_3_SnC_2_^14^, Ti_2_AlN deformed at 900 °C under gaseous confining pressure^15^ and slowly compressed Ti_3_AlC_2_ at 1200 °C^11^. Basal dislocations are prone to be arranged in arrays within the basal plane or walls along [0001]^9^. The accumulation of dislocation arrays within the basal plane gives rise to the bending of the basal plane, while that of dislocation walls leads to the formation of kinking boundaries.

Kinking boundaries in MAX phases are special tilt-dominated grain boundaries with limited twist components^8^,^9^. Farber *et al.* interpreted the kinking boundaries as dislocation walls with perfect dislocations threading along [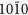]^8^, wherein the dislocations alternatively have a Burgers vector of 1/3[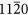] and 1/3[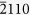]. An excess of one type of dislocation (e.g. 1/3[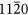]) over the other (e.g. 1/3[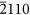]) contributes to the twist components^8^. Twist grain boundaries, on the contrary, can be regarded as screw dislocation networks^16^,^17^. For instance, small angle twist (sub-) grain boundaries (SATGBs) in the typical oxide ceramics Al_2_O_3_, (0001)/[0001], consist of hexagonal screw dislocation networks (HSDNs) with 1/3<
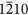
> basal dislocations^18^. The SATGBs in face- (with high stacking fault energy) and body-centered cubic metals comprise HSDNs as well^16^,^17^,^19^,^20^. In this letter, the dislocation segments of previously observed hexagonal networks in Ti_3_AlC_2_^11^ are determined to be screw type, and further, the formation and evolution of HSDNs/SATGBs are closely investigated.

## Results

### Formation of HSDNs

**Figure 1:**
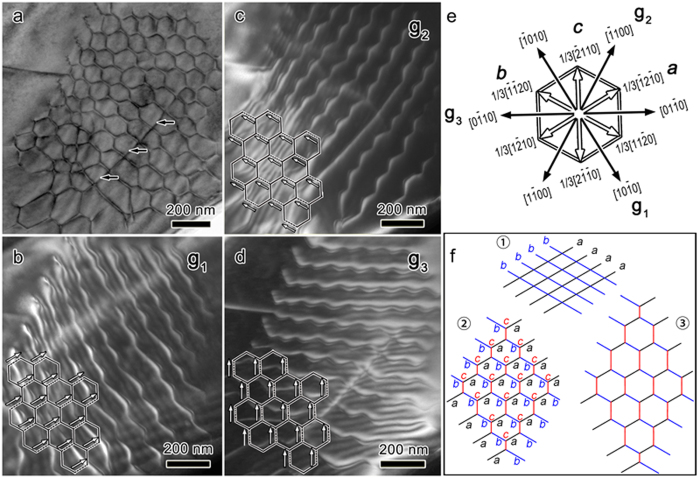
Formation of HSDNs. (**a**) A typical TEM morphology of HSDNs. Arrows denote unreacted dislocation segments. TEM dark field images are recorded with diffraction vectors (**b**) **g**_**1**_, (**c**) **g**_**2**_ and (**d**) **g**_**3**_. Solid and dotted lines illustrate the dislocations in and out of contrast, respectively. White arrows denote the Burgers vectors of the dislocation segments out of contrast. (**e**) Crystallographic directions of the diffraction vectors and Burgers vectors. (**f**) Illustrations of the formation of HSDNs. ***a***- (black) and ***b***-type (blue) dislocation arrays encounter (①), and react to form ***c***-type (red) dislocation segments (②). Dislocation configurations evolve to be HSDNs (③). Theoretically, the initial ***a***- and ***b***-type dislocation arrays can be screw, edge or mixed type. Nevertheless, irrespective of the nature of the initial dislocation arrays, the dislocation segments in the final equilibrium dislocation configuration are screw type. For the sake of simplicity, only the case for screw dislocation arrays is considered.

Basal dislocations in MAX phases can react to form complex dislocation networks and dense hexagonal cells^12^. In transmission electron microscopy (TEM) specimens sampled along directions both vertical and parallel to the load, hexagonal dislocation networks were frequently observed near the grain boundaries, wherein the dislocations are prone to be initiated or piled up. Figure 1a shows the typical TEM morphology. According to the **g·b** values in Table 1, the Burgers vectors of the dislocation segments out of contrast in Fig. 1b–d are 1/3[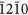], 1/3[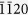] and 1/3[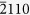] (denoted by ***a***, ***b*** and ***c***, Fig. 1e), respectively. For the sake of clarity, the dislocation segments in contrast are illustrated by solid lines and those out of contrast are marked with black dotted lines, as shown in Fig. 1b–d. White arrows represent the Burgers vectors of the dislocations out of contrast. Evidently, the Burgers vectors are parallel to the dislocation segments, indicating that the segments are screw type. The dislocation reaction at the nodes is ***a*** + ***b*** → ***c***. Figure 1f schematically illustrates the formation of HSDNs. The ***a***- and***b***-type dislocation arrays encounter each other and react to form ***c***-type segments. Under interaction forces, the final equilibrium configuration of HSDNs is established^16^. Theoretically, the initial ***a***- and ***b***-type dislocation arrays can be screw, edge or mixed type^16^, provided equation (1) is satisfied:


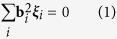


where **b**_*i*_ is the Burgers vector and **ξ**_*i*_ is the direction vector of dislocation line.

Since ***a***^2^ + ***b***^2^ > ***c***^2^, the above-mentioned reaction is energetically favorable. However, not all the crossing ***a***- and ***b***-type segments can react to form ***c***-type segments. As marked by the black arrows in Fig. 1a, a long ***a***-type segment fails to react with ***b***-type segments although they intersect at several positions, wherein the intersection angles are approximately 90°. This is more apparent in Supplementary Fig. S1. According to Hirth *et al.*, crossing dislocations that are a few degrees from being orthogonal cannot react^16^. Therefore, orthogons (Supplementary Fig. S1) or pentagons (Fig. 1a) instead of hexagons are formed because of unfavorable dislocation line directions. It is worth noting that, apart from ***a*** + ***b*** → ***c***(marked by the black circle in Supplementary Fig. S1), the reaction ***a*** + ***c*** → ***b***(marked by the red circle in Supplementary Fig. S1) contributes to the formation of HSDNs as well.

### The plane of twist sub-grain boundaries

**Figure 2:**
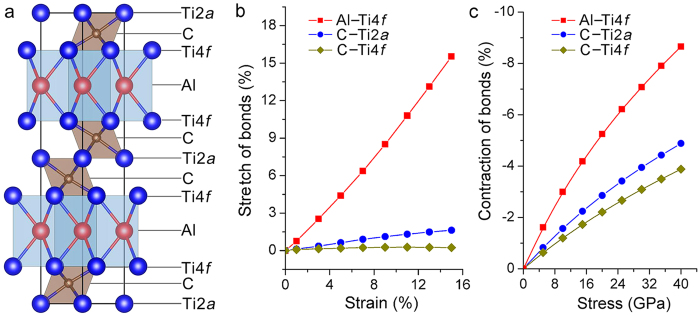
Changes in bond length. (**a**) Illustration of the unit cell of Ti_3_AlC_2_. Ti2*a* and Ti4*f* denote the Ti atoms located at the 2*a* and 4*f* Wyckoff sites of *P*6_3_/*mmc*, respectively. Changes in bond length of Al–Ti4*f*, C–Ti2*a* and C–Ti4*f* under (**b**) uniaxial tension along [0001], and (**c**) hydrostatic compression.

With the formation of HSDNs, SATGBs are established on the basal plane where the networks are located. Then, a scientific question naturally arises: which basal atomic plane is the most likely boundary plane? We address this issue via studies on atomic-scale deformation (illustrations are presented in Supplementary Fig. S2) and general stacking fault energy (GSFE) landscapes. The crystal structure of Ti_3_AlC_2_ comprises edge-sharing Ti_6_C octahedron layers bonded by C–Ti2*a* and C–Ti4*f*, and Ti_6_Al triangular prism layers bonded by Al–Ti4*f* (Fig. 2a, Ti2*a* and Ti4*f* denote the Ti atoms located at the 2*a* and 4*f* Wyckoff sites of *P*6_3_/*mmc*, respectively). Figure 2b plots the changes in bond length for Al–Ti4*f*, C–Ti2*a* and C–Ti4*f* against applied tensile strains along [0001]. It can be seen that the changes in C–Ti2*a* and C–Ti4*f* are negligible, and most of the strains are accommodated by the elongation of Al–Ti4*f*. Specifically, the stretches of Al–Ti4*f* are 6∼10 and 9∼66 times those of C–Ti2*a* and C–Ti4*f*, respectively. Similar features can be identified in the hydrostatic compression (Fig. 2c), where the contractions of Al–Ti4*f* are 2∼3 times those of C–Ti2*a* and C–Ti4*f*. Therefore, Al–Ti4*f* is the weakest bond in Ti_3_AlC_2_, and shear is believed to occur most easily therein.

**Figure 3:**
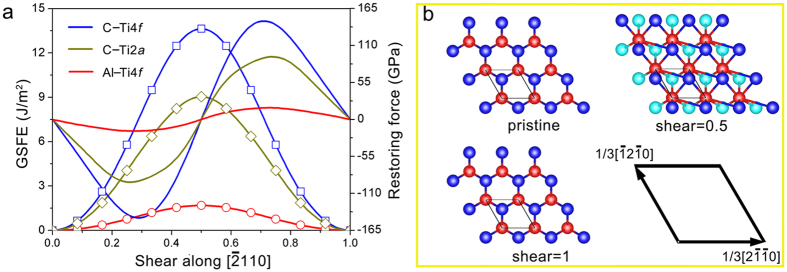
GSFE and restoring force. (**a**) GSFE (lines with symbols) and restoring force curves for shear deformations with breaking C–Ti4*f*, C–Ti2*a* and Al–Ti4*f*. The shear displacement is normalized by the length of 1/3[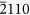]. (**b**) Atomic configurations of Ti_6_Al triangular prismatic layers for the shear deformation with breaking Al–Ti4*f*. Al atoms are denoted by red balls. Balls in cyan and blue highlight the Ti atoms just below and above the slip plane, respectively.

To further quantitatively confirm this from an energetic point view, GSFE was calculated (see the Methods and Supplementary Fig. S3 for details). The GSFE of Al–Ti4*f* is significantly lower than those of C–Ti4*f* and C–Ti2*a* (Fig. 3a), and the local maximum (USF) of Al–Ti4*f* is only 18.9% and 12.5% of those of C–Ti2*a* and C–Ti4*f*, respectively (Table 2). Besides, the ideal shear strength (maximum restoring force, defined as the maximum slope of the GSFE curve) of Al–Ti4*f* is only 19.2% and 12.2% of those of C–Ti2*a* and C–Ti4*f*, respectively (Table 2), which is consistent with the trend of interplanar spacing. Thereby, the screw dislocations are believed to be initiated by breaking the Al–Ti4*f* bonds^21^. Further, the SATGBs are most probably located between the Al and Ti4*f* atomic layers.

## Discussion

**Figure 4:**
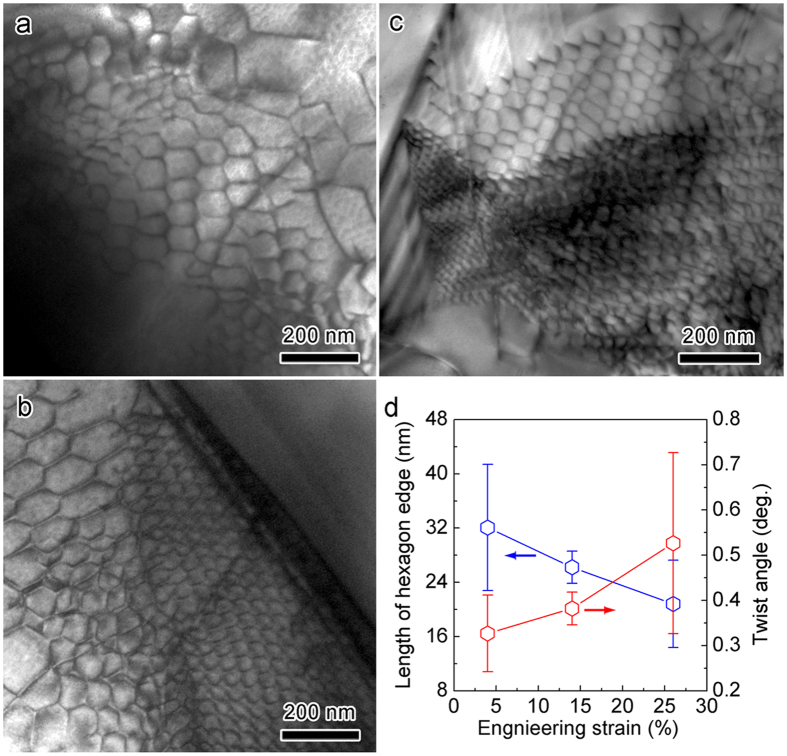
Evolution of twist angle with the deformation. Typical TEM morphologies of HSDNs in the sample deformed by (**a**) 4%, (**b**) 14% and (**c**) 26%. (**d**) Strain dependence of the length of hexagon edge and twist angle. For the sample with large deformations (14% or 26%), small HSDNs can be identified in the large networks.

MAX phases are well recognized to be of bonding anisotropy^3^,^22^,^23^,^24^,^25^: M_6_X octahedron layers are strongly covalently bonded, while adjacent M–X slabs are relatively weakly coupled by M–A metallic bonds. For Ti_3_AlC_2_, the ideal shear strength with breaking Al–Ti4*f* is only 19.2% and 12.2% of those with breaking C–Ti2*a* and C–Ti4*f*, respectively (Table 2), indicating that the most probable shear plane is the one between the Al and Ti4*f* layers^21^. Schematically, the shear of Ti_6_Al triangular prisms is illustrated in Fig. 3b. Therefore, the SATGBs observed in this study are believed to be there. As a characteristic parameter of the twist boundary, the twist angle, *θ*, can be estimated by equation (2)^16^,^20^:


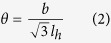


where *b* and *l*_*h*_are the lengths of the Burgers vector and hexagon edge, respectively. The calculated twist angle in Fig. 1a is approximately 0.26°. Figure 4a–c present the typical TEM morphologies of the HSDNs in the specimens with various deformations (4%, 14% and 26%). As the deformation proceeds, new dislocations intersect and react with the as-formed hexagonal networks. Consequently, small hexagonal dislocation cells form in large cells, and the average cell size diminishes. Statistical data of *l*_*h*_ and *θ* are plotted in Fig. 4d against the strain. It can be seen that the twist angle scales with the applied strains. For the sample deformed by 26%, the twist angle is about 0.5°. Notably, the HSDNs can be observed in other slowly deformed MAX phases (like Ti_2_AlC, Nb_4_AlC_3_ and *etc.*, Supplementary Fig. S4). The formation of HSDNs/SATGBs is generic to low-energy dislocation configurations in slowly deformed MAX phases. For MAX phases, the collective behavior of basal dislocations includes not only the previously reported accumulation of dislocations vertical and parallel to the basal plane^9^, but also the formation of HSDNs/SATGBs.

Dislocations are the carriers of plastic deformation. Their mutual interactions and reactions bring about work hardening. The formation of HSDNs contributes to previously identified strain hardening of Ti_3_AlC_2_^11^, giving rise to SATGBs. Since the twist angle is very small (around 0.5**°**), the contribution of SATGBs to the plastic deformation of Ti_3_AlC_2_ is quite limited. Sub-grains have been reported in the tensile and compressive creep of MAX phases^26^,^27^,^28^. Since the SATGBs are formed in compression with remarkably low strain rates, it is inferred that they likely contribute to the creep at high temperature.

With the formation of low energy dislocation structures, misorientations at grain and sub-grain scale are established. The misorientation angle increases with the strain in metals, linearly^29^,^30^ or in a power law (with an exponent of 1/2)^31^,^32^. Our work indicates that the twist angle scales with the deformation in a roughly linear manner in the investigated strain and temperature ranges.

In summary, small angle twist sub-grain boundaries (around 0.5°) have been ubiquitously observed in uniaxially compressed Ti_3_AlC_2_. The twist sub-grain boundaries predominantly comprise hexagonal screw dislocation networks that result from basal dislocation reactions. The grain boundary plane is believed to be between the relatively weakly bonded Al and Ti4*f* layers. In addition, it is unambiguously demonstrated that the twist angle scales with the deformation. This work may shed light on the formation of low-energy dislocation configurations and its evolution with the deformation of MAX phases.

## Methods

### High-temperature deformation

Ti_3_AlC_2_ bulk sample was synthesized using the method reported by Wang and Zhou^33^. For the compression tests, cylinders about 9 mm in diameter and 12 mm in height were cut from the as-prepared sample by electric discharge machining and then mechanically polished. Subsequently, three cylinders were compressed to a strain of 4%, 14% and 26% at 1200 °C with a strain rate of 10^−5^ s^−1^ in a universal testing machine (SANS CMT4204, Shenzhen, China).

### TEM characterization

Dislocation configurations were analyzed using a transmission electron microscope (FEI Tecnai G2 F20, Oregon, USA) working at 200 kV. For TEM investigations, slices were cut from the deformed sample in two directions, vertical and parallel to the load. Then, the slices were mechanically thinned and ion-milled to be electron transparent. The Burgers vectors of the dislocations were determined by the **g**·**b** method. For each deformation, statistics of hexagonal cell size were determined on seven TEM images obtained from different regions.

### First-principles calculations

The deformation at atomic scale was modelled with the CASTEP module^34^. Electronic exchange-correlation energy was treated under a generalized gradient approximation (GGA–PBE)^35^,^36^. Interactions of electrons with ion cores were represented by Vanderbilt-type ultrasoft pseudopotential^37^. The Broyden–Fletcher–Goldfarb–Shanno minimization method was used for geometry optimization, where the plane-wave cut-off energy and the Brillouin zone sampling were fixed at 450 eV and 5 × 5 × 2 Monkhorst–Pack-point meshes^38^, respectively. Differences in total energy, maximum ionic Hellmann-Feynman force, maximum ionic displacement and maximum stress were converged to 5 × 10^−6^ eV/atom, 0.01 eV/Å, 5 × 10^−4^ Å and 0.02 GPa, respectively. The fully optimized structure was used for uniaxial tension and hydrostatic compression. The deformation modes are illustrated in Supplementary Fig. S2. To ensure a uniaxial deformation along [0001], the lattice parameters perpendicular to the applied strain, as well as the internal coordinates of atoms, were fully relaxed until the stresses were converged to 0.02 GPa. For the GSFE calculation, a supercell with twenty-five atomic layers with Al layers at the center and surfaces was constructed using the fully optimized Ti_3_AlC_2_ unit cell, where a vacuum slab of 15 Å in thickness was symmetrically added (see Supplementary Fig. S3). The disregistry on the plane of interest was introduced by rigidly shifting all the atoms above the target plane relative to those below that plane along 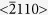. Without relaxing the supercell further, total energies of faulted structures were calculated. Then, the energy difference between the faulted and unfaulted structures gives the GSFE.

## Additional Information

**How to cite this article**: Zhang, H. *et al.* On the small angle twist sub-grain boundaries in Ti_3_AlC_2_. *Sci. Rep.* **6**, 23943; doi: 10.1038/srep23943 (2016).

## Supplementary Material

Supplementary Information

## Figures and Tables

**Table 1:**
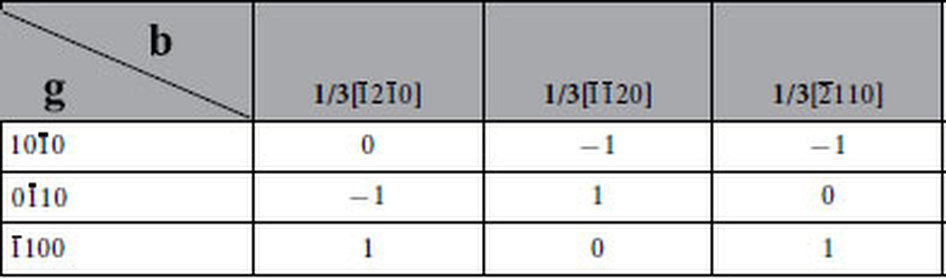
Values of g·b for basal dislocations in Ti_3_AlC_2_.

Since no stacking faults were observed in the present studies, only the Burgers vectors (b) of full dislocations are considered. Imaged with a diffraction vector g, dislocations with g·b = 0 are out of contrast.

**Table 2:** USF, ideal strength and interplanar spacing.

Bonds	USF (J/m^2^)	Ideal strength (GPa)	*d* (Å)
Al–Ti4*f*	1.7	17.9	2.26
C–Ti2*a*	9.0	93.4	1.07
C–Ti4*f*	13.6	146.6	1.31

USF is the local maximum of the GSFE curve. Ideal strength is defined as the maximum slope of the GSFE curve. *d* is interplanar spacing illustrated in Supplementary Fig. S3.
