# Supplementary material for: On the small angle twist sub-grain boundaries in Ti3AlC2
Source: Sci Rep. 2016 Apr 1;6:23943. doi: 10.1038/srep23943 (PMC4817052; doi:10.1038/srep23943)
Supplement: Supplementary Information [file srep23943-s1.pdf]

Supplementary Information

On the small angle twist sub-grain boundaries in Ti_3AlC_2

Hui Zhang[1,2], Chao Zhang[1], Tao Hu[1,2], Xun Zhan[3], Xiaohui Wang[1] &Yanchun Zhou[4]

[1]Shenyang National Laboratory for Materials Science, Institute of Metal Research, Chinese Academy of Sciences, 72 Wenhua Road, Shenyang 110016, China.
[2]University of Chinese Academy of Sciences, Beijing 100049, China.
[3]Department of Materials Science and Engineering, Case Western Reserve University, 10900 Euclid Ave, Cleveland, OH, 44106, USA.
[4]Science and Technology on Advanced Functional Composite Laboratory, Aerospace Research Institute of Materials & Processing Technology, No.1 South Dahongmen Road, Beijing 100076, China.

Correspondence and requests for materials should be addressed to X.H.W.. Email: wang@imr.ac.cn.

Supplementary Figures

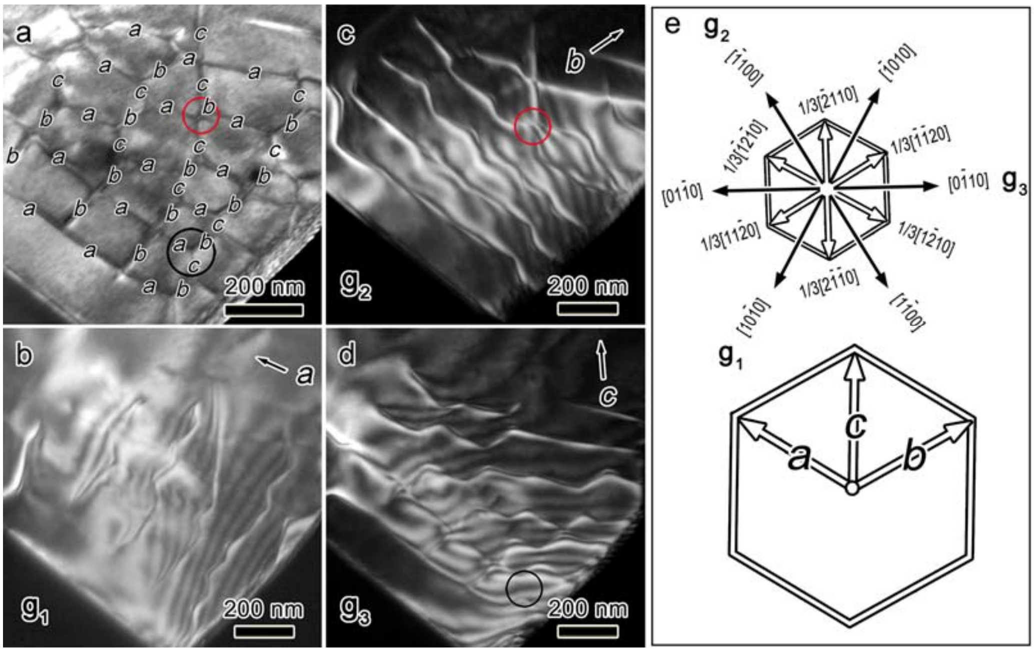

Figure S1. Transmission electron microscopy (TEM) morphologies of dislocation networks with orthogons and hexagons. (**a**) A typical TEM morphology of the dislocation networks. Burgers vectors of each dislocation segments are labeled by ***a***, ***b*** and ***c***. Black and red circles highlight dislocation reactions ***a*** + ***b*** → ***c*** and ***a*** + ***c*** → ***b***, respectively. TEM dark field morphologies were imaged with diffraction vectors (**b**) $\mathbf{g_1}$, (**c**) $\mathbf{g_2}$ and (**d**) $\mathbf{g_3}$. The Burgers vectors of the dislocation segments out of contrast are marked by arrows and denoted by ***a***, ***b*** and ***c***, respectively. (**e**) Illustrations of the crystallographic directions of diffraction vectors and Burgers vectors.

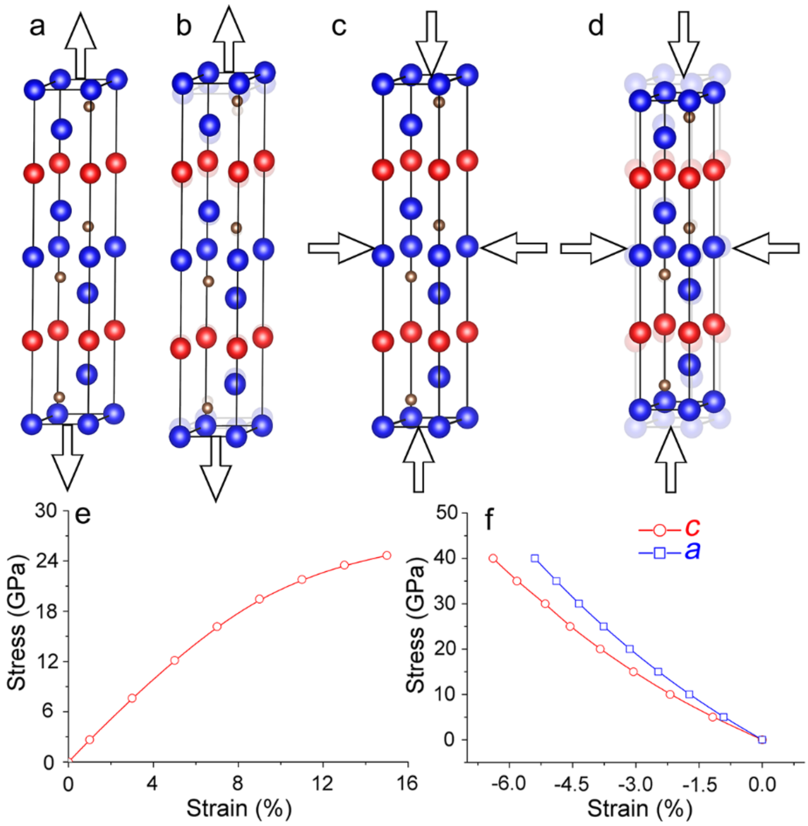

Figure S2. Illustrations of atomic-scale deformation and stress-strain curves. (**a,b**) Uniaxial tension. Ti, Al and C atoms are denoted by blue, red and brown balls, respectively. To ensure a uniaxial deformation along [0001], lattice parameters perpendicular to the applied strains, as well as internal coordinates of atoms, were fully relaxed until stresses were converged to 0.02 GPa. (**c,d**) Hydrostatic compression. The unit cell was fully optimized with prescribed hydrostatic pressures ranging from 0 to 40 GPa. **(e)** and (**f**) are the calculated stress-strain curves for the uniaxial tension and hydrostatic compression, respectively. Red and blue curves in (**f**) depict the stress evolution with the strain along ***c*** ([0001]) and ***a*** (1/3[$2\bar{1}\bar{1}0$]), respectively.

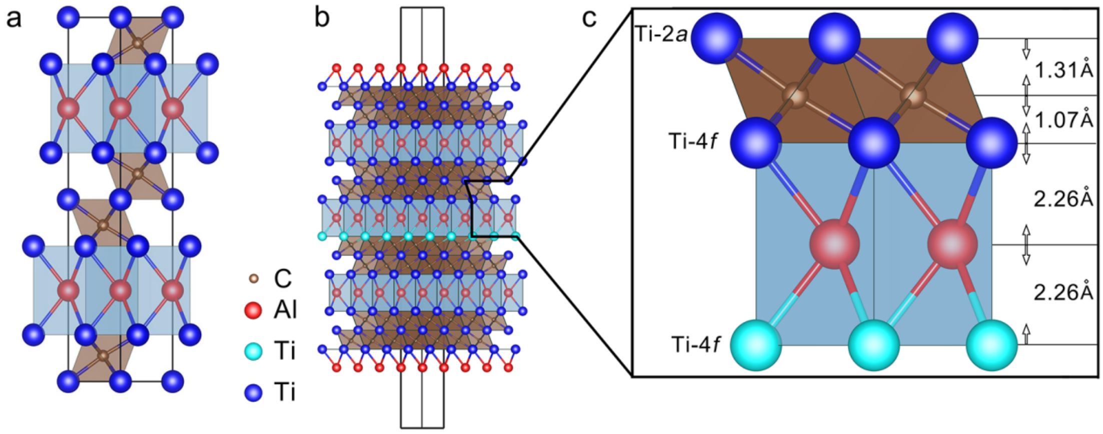

Figure S3. Illustrations of the model used for general stacking fault energy calculations. (**a**) Unit cell of Ti_3AlC_2. Edge-sharing Ti_6C octahedron layers are sandwiched by Ti_6Al triangular prisms. Ti, Al and C atoms are denoted by blue, red and brown balls, respectively. (**b**) Constructed supercell for general stacking fault energy calculations. Twenty-five atomic layers with an Al layer centered are involved. Balls in cyan denote the Ti atoms just below the central Al layer. (**c**) Illustration of the Ti_6C octahedrons and Ti_6Al triangular prisms. Interplanar spacings between C and Ti2*a*, C and Ti4*f*, and Al and Ti4*f* are 1.31, 1.07 and 2.26 Å, respectively. Ti2*a* and Ti4*f* denote the Ti atoms located at the 2*a* and 4*f* Wyckoff sites of $P6_3/mmc$, respectively.

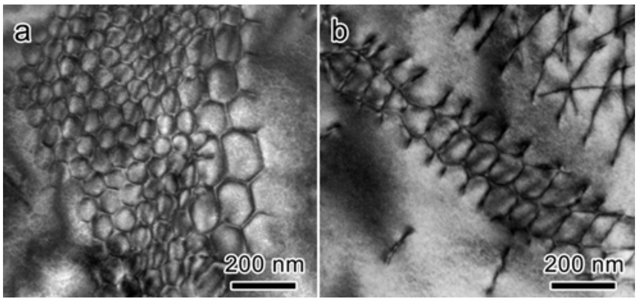

Figure S4. Hexagonal screw dislocation networks in Ti_2AlC and Nb_4AlC_3. Transmission electron microscopy morphologies of hexagonal screw dislocation networks in (**a**) Ti_2AlC uniaxially compressed at 1200 °C with a strain rate of 10^{-5} s^{-1}, (**b**) Nb_4AlC_3 uniaxially compressed at 1400 °C with a strain rate of 10^{-4} s^{-1}. Since Nb_4AlC_3 has a high resistance to deformation (with a less than 4% plastic strain), hexagonal screw dislocation networks in Nb_4AlC_3 are not as obvious as those in Ti_2AlC and Ti_3AlC_2.
